# Acknowledgment of Reviewers

**DOI:** 10.1128/mBio.02303-16

**Published:** 2016-12-30

**Authors:** Arturo Casadevall

## EDITORIAL

On behalf of the editors of *mBio*, I gratefully acknowledge the following individuals, who served as reviewers for the journal in 2016. Their time and effort in reviewing articles have been essential in ensuring the high quality of our publications, and their help is greatly appreciated.

Jesus Aguirre

Brian Ahmer

Sebastian Alberti

Luke Alderwick

Bree Aldridge

Maria Theresa Alera

Gladys Alexandre

Caitilyn Allen

Lee-Ann Allen

Rudolf Amann

Gaya Amarasinghe

Peter Andersen

Sandra Andersen

Siv Andersson

David Andes

Leonardo Andrade

Cristel Archambaud

Michelle Arnold

David Aronoff

Alexander Askenov

Taj Azarian

Andreas Bäumler

Yong-Sun Bahn

John Baines

Mitchell Balish

Terry Ball

Alan Barrett

Michael Bartlett

Anthony Baughn

Clifford Beall

Avraham Beer

Marcel Behr

Daniel Beiting

Mar Benavides

Stephen Beres

Howard Berg

Eric Bergeron

Arnold Berk

Kristen Bernard

Thomas Bernhardt

Lionel Berthoux

Francesco Berti

Paul Berube

Debra Bessen

Bruce Beutler

Tanmoy Bhattacharya

Jennifer Biddle

Markus Bischoff

Russell Bishop

David Blair

Steven Blanke

Jesus Blazquez

Brendan Bohannan

Alberto Bosque

Daria Bottai

Cara Boutte

Ethna Boyd

Salvatore Bozzaro

Patricia Bradford

Maria Brandl

Mya Breitbart

Nichole Broderick

Dennis Brown

Melissa Brown

Glenn Browning

Robert Brunham

Juliane Bubeck Wardenburg

Carmen Buchrieser

Christopher Buck

Dirk Bumann

David Bundle

Lori Burrows

Alessia Buscaino

David Bzik

Jeffrey Cameron

Isaac Cann

Chelsea J. Carey

Yehuda Carmeli

Daniel J. Carr

Glen Carter

Josep Casadesus

Jean Celli

Trinad Chakraborty

Mathias Chamaillard

Henry Chambers

Patricia Champion

Eugene Chang

Jeffrey Chen

Rubing Chen

Caroline Chenard

Yury Chernoff

Ludmila Chistoserdova

Peter Christie

Paul Clapham

Paul Cohen

Anamaris Colberg-Poley

Mattias Collin

Laurie Comstock

Andrew Conlan

Gregory Cook

Peter Cook

Roland Cooper

Pierre Cornelis

Timothy Cover

Leah Cowen

Carolyn Coyne

Alister Craig

Alan Cross

Ajai Dandekar

Cristian Danna

Pranav Danthi

Siddhartha Das

Sean Davies

Dana Davis

Ian Davis

Piet de Boer

Piet de Groot

Alexandre de Menezes

Kirk Deitsch

Maurizio Del Poeta

Frank DeLeo

John Demain-Sauer

Donald Demuth

Tanvi Dhere

Felipe Diaz-Griffero

Gregory Dick

Thomas Dick

Binh Diep

Stephen Diggle

Rod Dillon

Victor DiRita

Larry Dishaw

Maziar Divangahi

Jeremy Dodsworth

Yohei Doi

Esteban Domingo

Robert Doms

Charles Dorman

Jorn Dunkel

Dan Dwyer

Leo Eberl

Bernhard Ehlers

Garth Ehrlich

Sabine Ehrt

Jonathan Eisen

Amie Eisfeld-Fenny

David Engelthaler

John Erb Downward

Robert Ernst

Tara Essock-Burns

Ana Eulalio

Matthew Evans

Oliver Fackler

Daniel Falush

Michael Federle

Heinz Feldman

Michael Ferdig

Thomas Ferenci

Ana Fernandez-Sesma

Paul Fey

Noah Fierer

Sergio Filipe

Alain Filloux

Klas Flardh

Ervin Fodor

Anja Forche

Karl Forchhammer

James Forrest

Naomi Forrester

Ron Fouchier

Betsy Foxman

Gregory Francius

Karen Frank

James Fraser

Jorge Frias-Lopez

W. Florian Fricke

Marc Fuchs

Robert Fujinami

Clay Fuqua

Anthony Gaca

Juan Carlos Galan

Michael Gale, Jr.

Richard Gallo

Yunn-Hwen Gan

Roman Ganta

Feng Gao

Adolfo García-Sastre

Jennifer Gardy

Melissa Garren

Danielle Garsin

Angie Gelli

Caroline Genco

Martin Gengenbacher

Stephane Genin

Nicole Gerardo

Gunther Gerisch

Mahmoud Ghannoum

Jason Gigley

Tim Gilberger

Scott Gilbert

Steven Gill

Michael Gillings

Zemer Gitai

Pierre Gladieux

N. Louise Glass

Britt Glausinger

Matthew Goddard

Rajesh Gokhale

Gustavo Goldman

Tatyana Golovkina

Tania Gomes

Michael Good

Andrew Goodman

Paul Gorry

Heinrich Gottlinger

Friedrich Götz

Sylvain Goutelle

Jeffrey Gralnick

Lone Gram

Thomas Gramberg

Peter Graumann

Carol Gross

Dennis Gross

Yang Gu

Susanne Häussler

Abderrahman Hachani

Ted Hackstadt

Max Haggblom

Anders Hakansson

Mohamed Hakimi

Kasturi Haldar

Benjamin Hale

Jason Hall

Pamela Hall

Lynn Hancock

Steven Harris

Graham Hatfull

Vasili Hauryiuk

Sheng He

Nicholas Heaton

Johannes Hegemann

Jens Heller

John Helmann

Crystal Hepp

Anat Herkskovit

Johannes Herrmann

Anat Herskovits

Heather Hickman

Ann Hill

Russell Hill

Markus Hilty

Peter Hinterdorfer

Jay Hinton

Robert Hirt

Deborah Hogan

Brenda Hogue

Johanna Holm

Richard Honkanen

Stacy Horner

Benjamin A. Horwitz

Jonathan Howard

Guanghua Huang

Haibin Huang

Kerwyn Huang

Grant L. Hughes

Raymond Hui

David Hunstad

Julian Hurdle

Zoya Ignatova

Oleg Igoshin

Renato Iozzo

William Jackson

Ursula Jakobs

Amanda Jamieson

Yu-Sin Jang

Grant Jensen

Xiao Jie

Shouguang Jin

Timothy Johnson

David Johnston

Stipan Jonjic

Jonathan Juliano

Glenn Kaatz

David Kadosh

Dale Kaiser

Marina Kalyuzhnaya

Jeremy Kamil

Bavesh Kana

Tatsuo Kanda

Melissa Kane

Stefan Kappe

Stephanie Karst

Kazuyuki Kasahara

Fatah Kashanchi

Yoshihiro Kawaoka

Nancy Kedersha

Thomas Kehl-Fie

Chris Kempes

Melissa Kendall

Shabaana Khader

Stephen Kidd

Patricia Kiley

Minsu Kim

Samantha King

Frank Kirchhoff

David Kirchman

Hannah Klein

David Knipe

Laura Knoll

Ben Knowles

Julia Koehler

Andrew Koh

Arash Komeili

James Konopka

Manfred Kopf

Anita Koshy

Ákos T. Kovács

Susan Koval

Barry Kreiswirth

Gopinath Krishnamoorthy

Christopher Kristich

Mitch Kronenberg

Damian Krysan

Meta Kuehn

Jens Kuhn

Richard Kuhn

Stefan Kunz

Andreas Kupz

Edo Kussell

Nikos Kyrpides

Meytal Landau

Herrero Lara

Adam Lauring

Thomas Lavstsen

Ramanan Laxminarayan

Helen Lazear

Elizabeth Leadbetter

Jared Leadbetter

Francois Lebreton

Maryse Lebrun

Claude Leclerc

Chia Lee

Joungmin Lee

Seon-Woo Lee

Kevin Legge

Julian Leibowitz

John Leong

Cammie Lesser

Michael Lesser

François Leulier

Kim Lewis

Chen Liang

Michelle Liberton

Susan Liebman

Jean Lim

Andrew Limper

Yee-Shin Lin

Stephen Lindemann

Lisa Lindesmith

Jodi Lindsay

Jiqiang Ling

Frederique Lisacek

George Liu

Jose Lopez-Ribot

Benjamin Loppin

Stephen Lory

Arnold Louie

Petra Louis

Steven Lower

Ting Lu

Peter Lund

Aldons Lusis

Joseph Lutkenhaus

Erich Mackow

Hiten Madhani

Eshwar Mahenthiralingam

Martha Malapi-Wight

Mark Mandel

Michael Manson

Costas Maranas

Jon Marles-Wright

Mark Marsh

Eric Martens

Rowena Martin

Jose Martinez

Vega Masignani

Revati Masilamani

Kevin Mason

Michael Maurizi

Didier Mazel

Sarkis Mazmanian

Andrew McBain

Shonna McBride

Linda McCarter

John McCormick

Mary McDowell

Quinn McFrederick

Kevin McIver

James McKinlay

Jason McLellan

Alan McNally

Didier Menard

Seppo Meri

Houra Merrikh

Ilhem Messaoudi

Jessica Metcalf

Lisbeth Meunier-Goddik

Shirley Micallef

Thomas Michiels

Tam Mignot

Stephen Miller

Abraham Minsky

Dominique Missiakas

Tim Mitchell

Harry Mobley

Robert Modlin

Christopher Mody

Wiebke Mohr

Denise Monack

Ruth Montgomery

Guido Mora

Iain Morgan

Lynn Morris

Mark Morrison

Joachim Morschhäuser

Donald Mosier

Karl Munger

Timothy Murphy

Barbara Murray

Kristy Murray

Karin Musier-Forsyth

Joe Mymryk

David Myrold

Carey Nadell

Hiroki Nagai

Roger Narayan

Xavier Nassif

H. Nauwynck

David Nelson

Daling Ning

Derek Nolan

Minou Nowrousian

Eric Nuermberger

Joshua Obar

Amanda Oglesby-Sherrouse

Anil Ojha

Fabiano Oliveira

Michal Olszewski

Akira Ono

Carlos Orihuela

George O'Toole

Melanie Ott

Michael Otto

J.-H. Ou

Julie Overbaugh

Cara Pager

Tracy Palmer

Timothy Palzkill

Rebecca Parales

Kristin Parent

William Parks

John Patton

Norman Pavelka

Andrew Pekosz

Vladimir Pelicic

Philip Pellett

Xuanxian Peng

Ines Pereira

John Perfect

Eva Pericolini

David Perlin

Stanley Perlman

Steve Perlman

Andreas Peschel

Constantinos Petrovas

Julie Pfeiffer

Andrew Phipps

Sacha Pidot

David Pignol

Dylan Pillai

Johann Pitout

Jacqueline Plumbridge

Mechthild Pohlschroder

Alessandra Polissi

Katherine Pollard

Stephen Porcella

Alexa Pragman

Robert Quivey

Manuela Raffatellu

Paul Rainey

Seth Rakoff-Nahoum

Kumaran Ramamurthi

Jayne Raper

David Rasko

Angela Rasmussen

Will Ratcliff

Julian Rayner

Andrew Read

Timothy Read

Larry Reitzer

Jyothi Rengarajan

Michelle Reniere

Federico Rey

Andrew Rice

Anthony Richardson

Rebeca Rico-Hesse

Kristian Riesbeck

Arne Rietsch

Kim Ritchie

Adam Roberts

Eduardo Rocha

Richard Roden

Gloria Rodriguez

Francisco Rodriguez-Valera

Paul Roepe

Morgane Rolland

R. Roop

Warren Rose

Amy Rosenfeld

Alan Rothman

Craig Roy

Seth Rubin

David Rudner

Natividad Ruiz

Jacob Russell

Alexey Ruzin

Jeroen Saeij

Selena Sagan

Jason Sahl

Padmini Salgame

John Samuelson

Maria Sandkvist

Helene Sanfacon

Álvaro San Millán

Martin Sapp

Alain Sarniguet

Peter Sarnow

Daniel Sauter

Isabelle Schalk

Jeffrey Schertzer

Enrico Schleiff

Patrick Schlievert

Susan Schlimpert

Patrick Schloss

Jonas Schluter

Scott Schmid

Dirk Schnappinger

Dean Scholl

Sabrina Schreiner

Anthony Schryvers

Heinrich Schulenberg

Michael Schurr

Martin Schuster

Patrick Seed

Anna Seekatz

Leopoldo Segal

Julia Segre

H. Seifert

Peter Setlow

Ethan Settembre

Stephanie Seveau

Reed Shabman

Ashley Shade

William Shafer

Mara Shainheit

Yousif Shamoo

Donald Sheppard

Louis Sherman

Barbara Sherry

Frank Shewmaker

Pei-Yong Shi

Sunny Shin

Mari Shinohara

Natalie Silmon de Monerri

Jared Silverman

Tony Sinai

Steven Singer

Brajesh Singh

Eric Skaar

James Slauch

Mark Slifka

Kornelia Smalla

James Smith

Greg Somerville

Joseph Sorg

Alexandra Soukup

Victor Sourjik

Renato Souza

Stephen Spiro

Alfred Spormann

Michael Springer

Gary Stacey

Jason Stajich

Jack Stapleton

Fabrizia Stavru

Chad Steele

James Stegen

Magnus Steigedal

Lisa Stein

Silke Stertz

Frank Stewart

George Stewart

Scott Stibitz

Timothy Stinear

Joseph Strauss

Patrick Stuart

Xiaolei Su

Xin-Zhuan Su

Garret Suen

Paul Sullam

William T. Sullivan

Paul Sumby

Anne Summers

Keer Sun

Yvonne Sun

Ivan Surovtsev

Mehul Suthar

Kelly Swanson

Edward Swords

Michiko Taga

Adel Talaat

Hengli Tang

Julien Tap

Luis Teixeira

Ben Temperton

Max Teplitski

Carolyn Teschke

Andreas Teske

Robert Thacker

Kevin Theis

Casey Theriot

Volker Thiel

Linda Thomashow

J. Cameron Thrash

Scott Tibbetts

Mark Toleman

Marcelo Tolmasky

Hung Ton-That

Edward Topp

Bryan Tracy

M. Trent

Ralph Tripp

Krzysztof Trzciński

David Tscharke

Christian Tschudi

Jeffery Twiss

Peter Uetz

Mary Valcanis

Raphael Valdivia

Peter Valentin-Weigand

Adrianus van der Velden

Patrick Van Dijck

Cecile van Els

Peter van Endert

Luc Van Kaer

Julia van Kessel

Willem van Schaik

Daria Van Tyne

Russell Vance

Carin Vanderpool

Yoshiki Vazquez-Baeza

Jan-Willem Veening

Greg Velcier

Vijayakumar Velu

Vittorio Venturi

Gayathri Vijayakumar

Joe Vogel

Waldemar Vollmer

Julia Vorholt

Daniel Voth

Grzegorz Węgrzyn

Joseph Wade

Samuel Wagner

Mats Wahlgren

Seth Walk

Christopher Walker

Mark Walker

Ross Waller

Robert Wallis

Timothy Walsh

Jens Walter

Jue Wang

Nian Wang

Tian Wang

Yue Wang

Ronald Washburn

Sing-Sing Way

Richard Webby

Friedemann Weber

Gunter Wegener

Christopher Weidenmaier

Joern-Hendrik Weitkamp

Matthew Weitzman

Paula Welander

David Weller

Hannah Wexler

Aaron White

John White

Michael Whitt

Kevin Wiehe

Lothar Wieler

Darin Wiesner

H. Wiley

Brian Wilkinson

Peter Williamson

Benjamin Willing

David Wilson

Joyce Wilson

Mary Wilson

Ned Wingreen

Wade Winkler

Sebastian Winter

Alan Wolfe

Benjamin Wolfe

Floyd Wormley, Jr.

Daniel Wozniak

Meredith Wright

Joao Xavier

Karina Xavier

Zhiyong Xi

Chaoyang Xue

Jinjuan Yan

Ching-Hong Yang

George Yap

Carl Yeoman

Joanne Yew

Hesna Yigit

Fitnat Yildiz

Shixue Yin

Kevin Young

Xi Yu

Nicholas Zachos

Thomas Zahrt

Einat Zchori-Fein

Jing-Ren Zhang

Lian-Hui Zhang

Youfu Zhou

Jun Zhu

John Ziebuhr

Wilma Ziebuhr

